# How abortion restrictions compromise progress in interventional maternal‐fetal research

**DOI:** 10.1002/pmf2.70095

**Published:** 2025-09-03

**Authors:** Emma Canepa, Lori Freedman, Asha N. Talati, Teresa N. Sparks, Nuriye Sahin Hodoglugil, Juan Gonzalez‐Velez, Julia E. H. Brown

**Affiliations:** ^1^ Center for Maternal‐Fetal Precision Medicine UCSF San Francisco California USA; ^2^ Pediatric Department of Surgery UCSF San Francisco California USA; ^3^ Advancing New Standards in Reproductive Health UCSF San Francisco California USA; ^4^ Bixby Center for Global Reproductive Health UCSF San Francisco California USA; ^5^ Department of Obstetrics & Gynecology University of North Carolina Chapel Hill North Carolina USA; ^6^ Department of Genetics University of North Carolina Chapel Hill North Carolina USA; ^7^ Department of Obstetrics & Gynecology UCSF San Francisco California USA; ^8^ Department of Humanities and Social Sciences UCSF San Francisco California USA

## 1 INTRODUCTION

Experimental maternal‐fetal interventions, including surgical and medical therapies, present families facing severe fetal diagnoses with the chance to improve outcomes and advance maternal, fetal, and neonatal care. However, inherent risks of pregnancy (like preterm labor, PPROM, hemorrhage, preeclampsia, and chorioamnionitis) are increased both by fetal anomalies and maternal‐fetal interventions. While these events are rare (1%–20% per procedure), dilation and evacuation or induction of labor are sometimes required to protect maternal life and health. As a result of the *Dobbs* decision removing federal abortion protections, clinicians must consider pre‐viability restrictions in 22 states as of May 2025 [1]. A total of 12 states ban abortion entirely. Lack of guidance from policymakers or regulatory authorities shifts responsibility for ethical practice in the context of restrictions onto others, including participants and researchers, with serious consequences. This opinion discusses clinical and ethical implications of abortion restrictions on maternal‐fetal research across abortion‐restrictive and abortion‐permissive states. We highlight how abortion restrictions fail to account for the complex reasons that participants in maternal‐fetal research may require an abortion for preservation of maternal life and health and hinder researchers’ abilities to provide safe, ethical participation in clinical trials. These restrictions limit comprehensive and full‐spectrum reproductive care and add impediments to the forward progress of innovative therapies for maternal‐fetal medicine.

## 2 HOW ABORTION RESTRICTIONS NEGATIVELY IMPACT THE LANDSCAPE OF MATERNAL‐FETAL RESEARCH

Due to the specialized nature and limited availability of maternal‐fetal treatment centers and relevant clinical trials, pregnant individuals interested in maternal‐fetal studies will often travel to other states to participate. Requirements for travel between the research site and the participant's home can range from one trip to a month‐long temporary relocation. Regardless of where a maternal participant is, a clinical scenario in which termination of pregnancy is recommended may arise. Many participants travel between states with inconsistent access to abortion, increasing concerns of maternal safety and medical liability.

### 2.1 Increased risk to maternal participants and providers

Enrollment in most interventional maternal‐fetal research occurs after the first trimester, well after the gestational age cut‐off in many states. Several major maternal‐fetal treatment centers are in states that impose significant restrictions on abortion, leaving pregnant participants with inadequate local access to abortion and the risk of significant travel to receive appropriate care. Abortion restrictions and their effects on medical practice compromise how participants in restrictive states navigate post‐procedure adverse events. In some abortion‐restrictive states, providers can no longer offer abortion even to preserve the pregnant patient's health [2]. Participants in abortion‐restrictive localities may have to wait medically unsafe amounts of time, until an event becomes life‐threatening, to receive care, if they receive care at all. They may suffer substandard treatment with higher risks than pregnancy termination, as some providers in abortion‐restrictive states perform caesarean sections when dilation and evacuation would be more appropriate [3]. Increased odds of maternal morbidity during pregnancy have already been seen in Texas since its 6‐week abortion ban went into effect in 2021 [2].

False claims that progress in maternal‐fetal therapy makes abortion obsolete [4, 5] overlook that most maternal‐fetal interventions are experimental and conditionally available. A given fetal diagnosis and intervention may have varied prognoses and results. For example, a woman carrying a previable fetus diagnosed with Sly syndrome who wants to enroll in a clinical trial may develop mirror syndrome from non‐immune hydrops fetalis. This could lead to a life‐threatening maternal hypertensive crisis, prohibit enrollment in a trial, and indicate the need for an abortion to preserve maternal life and health. Should a pregnant person choose to undergo previable fetoscopic surgery, there is increased risk for an intra‐amniotic infection, which would again require termination of pregnancy. As has been reported in multiple news stories since *Dobbs*, lack of abortion access in these situations can lead to tragic impacts on maternal life and health [2].

Clinicians in abortion‐restrictive states risk loss of licensure, felony charges, and imprisonment for providing abortions. While some bans include exceptions (i.e., “life of the mother”), they are rarely utilized as physicians cite ambiguity over when protections would apply. Maternal‐fetal researchers may face legal and professional risks should a participant experience an adverse pregnancy outcome while on‐study, either from providing a necessary abortion or if a fetal loss related to their research intervention occurs [6]. Abortion restrictions impeding standard obstetric practice may also increase the rate of serious adverse events in interventional maternal‐fetal research, impairing progress in developing novel therapies. This infringement on practice is further causing restrictive states to lose doctors trained in MFM and OBGYN, compounding risks to pregnant women and remaining clinicians [7].

### 2.2 Undermining of ethical practice

Increased maternal risks and prohibition of standard pregnancy management due to abortion restrictions raise ethical concerns about the processes of maternal‐fetal research, including questions of non‐maleficence and challenges to informed consent. In ethical clinical and research practice, physicians are obligated to “do no harm,” and participants should expect to receive medically appropriate care in the event of adverse pregnancy events during study participation [8]. When abortion access is limited, researchers are unable to guarantee access to standard obstetric care and cannot meet ethical standards for non‐maleficence in research practice. This impacts the risk‐benefit analysis in maternal‐fetal research, increasing thresholds for acceptable risk when participants lack access to abortion, challenging researchers’ ability to protect participants from harm.

If abortion is not available as an option when a fetal condition amenable to maternal‐fetal therapy is diagnosed, informed consent can be compromised. A key pillar of informed consent includes voluntary participation without undue influence and an understanding of all other options [8]. Therefore, consent discussions in maternal‐fetal research should involve nondirective counseling prior to participation, including review of all pregnancy options: maternal‐fetal intervention; continuation of the pregnancy with an unbiased explanation of the diagnosis; or termination of pregnancy. Without access to abortion, especially if a woman is not willingly pregnant, maternal‐fetal research could be considered coercive and the potential for therapeutic misconception is high [9], particularly in states where providers feel restricted in their ability to discuss or refer for termination procedures.

## 3 MATERNAL‐FETAL THERAPY IN ABORTION‐RESTRICTIVE VERSUS ABORTION‐PERMISSIVE STATES

The removal of one choice (abortion) should not necessarily result in further removal of choices (maternal‐fetal research). However, the current restrictive climate pressures maternal‐fetal researchers to develop strategies to ensure their work continues as safely and ethically as possible, stretching already overburdened research teams. These strategies may differ for those working in abortion‐permissive and abortion‐restrictive states.

### 3.1 Impact on scientific integrity and progress in abortion‐permissive states

For maternal‐fetal researchers in abortion‐permissive states, enrollment from abortion‐restrictive ones may be necessary to reach enrollment goals, especially in rare diseases. Participants navigating abortion restrictions require additional support, particularly when they have traveled away from their local institutions and resources. If a potential participant has questions about where they can access abortion, researchers must be prepared to walk them through their options locally and in their home state. Maintaining standard nondirective counseling practices means also accounting for logistical, financial, and institutional supports for providing an abortion should a participant arrive for consultation and choose to terminate.

Depending on aiding and abetting or travel restrictions, legal counsel or protection may be required. Reporting from May 2025 revealed that authorities in Texas used over 83,000 automatic license plate reader cameras across both restrictive and non‐restrictive states in search of a woman they claimed had self‐managed an abortion. As travel restrictions increase, these practices may become more common [10]. Financial support for participants to relocate and stay near the study site through treatment and delivery is ideal but not always feasible. Researchers must develop a safety plan if participants are sent home to an abortion‐restrictive state during the study, including confirming that a local team can provide timely care at all gestational ages if complications arise. This is a near‐impossible feat for researchers to understand and navigate the local landscape, practice, referral system, and geography of another state. Budgets and IRB applications may need to include reimbursement for travel, accommodations, and, perhaps from separate funding for social supports, the termination procedure itself for those who cannot otherwise access the full range of obstetric care.

### 3.2 Impact on scientific integrity and progress in abortion‐restrictive states

For fetal researchers in abortion‐restrictive states, it is important to clearly delineate the additional risks resulting from these laws during the informed consent process. With the legal landscape of abortion evolving quickly, multiple consent discussions and assessments about how access may impact participant safety may be necessary. Aligning care with ACOG guidelines will be challenging for providers in abortion‐restrictive states, where they may face penalties for even discussing termination procedures. Abortion care often requires time‐consuming conversations with hospital lawyers and administrators to determine circumstances where their hospital would support health‐ and lifesaving terminations. Concern over legality can take precedence over physician expertise [2].

Significant institutional and infrastructural supports are needed to mitigate maternal risks during clinical trial participation. Researchers may assess providing financial support for participants to relocate to their institution for the duration of the pregnancy to ensure immediate access to full‐spectrum care. If local restrictions challenge standard of care, researchers may consider only including gestational ages that they can promptly treat. If local laws and the participant's condition allow, funding may be needed to support the participant's travel to a state where they could receive an abortion. Finally, some researchers may consider pausing interventional maternal‐fetal research projects due to potential legal and professional consequences, should an intervention result in a fetal demise or complications requiring a termination procedure [6]. Such outcomes slow the pace of innovative and practice‐changing research and could adversely impact maternal and fetal health.

## 4 WHAT MATERNAL‐FETAL RESEARCHERS CAN DO TODAY

Maternal‐fetal researchers in abortion‐permissive and ‐restrictive states are struggling to navigate the current legal and political landscape of abortion access for their participants. Available tangible actions may differ by locality, institution, and available resources. Below, we list immediate actions maternal‐fetal researchers can consider taking to protect their participants:

- Stay informed of local abortion restrictions and available informational, legal, financial, and logistical abortion support (e.g., INeedAnA.com). News alerts or Substack Newsletters like Jessica Valenti's *Abortion, Every Day* can help track state and federal developments.
- Update consent forms and discussions to include potential pregnancy complications and resources available to treat them (including abortion services), whether or not they might occur as a result of participation in the clinical trial. If relevant to your site, consider discussing that lack of access to abortion may increase maternal risk should complications occur. Explore initiating similar discussions with your local IRB or ethics board.
- Maintain standard, nondirective counseling during all informed consent discussions.
- Consider additional funding sources for participant relocation to the study site. Additional funds may also be considered to ensure full coverage of study‐related costs and care for pregnancy complications to mitigate widening disparities in clinical trial and abortion access.
- Perform safety assessments for and plans with participants who must return to abortion‐restrictive states (or all participants if the site is in an abortion‐restrictive state) in the event of a pregnancy complication. Safety assessments and planning may include reviewing the distance of the participant's home to a hospital equipped to care for complex pregnancy cases, participant's financial and social resources for travel to such a hospital, or creating an emergency response protocol with the participant and local care teams should pregnancy complications arise.
- Ensure maternal participants can easily contact the research team and access appropriate medical counselling if they experience symptoms suggestive of a pregnancy complication.
- Educate about how abortion restrictions negatively impact interventional maternal‐fetal research by documenting and presenting experiences to professional societies (SMFM, ACOG, Society of Family Planning), colleagues, policymakers, hospital administrators, and the broader community.
- Document harm caused to patients and research participants as a result of abortion restrictions.
- Consider advocating for policy changes to expand access to and protection for abortion at every level: within your institution, county, state, or federally. If you have bandwidth, speak at public hearings, meet with lawmakers, or engage with journalists to share your expertise. Researchers and practitioners who understand the intricacies of and experience firsthand the negative impacts of abortion restrictions can have a major impact on policymakers and public opinion.

## 5 CONCLUSION

Abortion has always played a critical role in the care of pregnant patients, including research participants. Interventional maternal‐fetal research without access to abortion raises concerns about maternal safety, ethical research practices, and the future of maternal‐fetal therapy. Maternal‐fetal researchers across the United States are burdened with navigating restrictions as they attempt to make progress in the field while protecting their participants. Abortion bans and restrictions overlook the complexities of pregnancy, maternal‐fetal care, and research, threatening researchers’ ability to safely care for their participants. All who are invested in fetal and maternal health must confront one of the many unintentional harmful realities of abortion restrictions: slowing, obstructing, and in some cases, halting lifesaving research in maternal‐fetal medicine.

## CONFLICT OF INTEREST STATEMENT

The authors declare no conflicts of interest.

## ETHICS STATEMENT

This commentary does not involve any studies with human participants or animals performed by the authors. Therefore, ethical approval and informed consent were not required.

## Data Availability

Data sharing is not applicable to this article as no datasets were generated or analyzed during the current study.
